# Nomogram to predict 3 month prognosis of acute ischemic stroke among young adults

**DOI:** 10.3389/fneur.2024.1487248

**Published:** 2025-01-30

**Authors:** Qian He, Miaoran Wang, Haoyue Zhu, Ying Xiao, Rui Wen, Xiaoqing Liu, Yangdi Shi, Linzhi Zhang, Bing Xu

**Affiliations:** ^1^Qionglai Traditional Chinese Medicine Hospital, Chengdu, China; ^2^Shenyang Tenth People’s Hospital (Shenyang Chest Hospital), Shenyang, China; ^3^China Medical University, Shenyang, China; ^4^Shenyang First People’s Hospital, Shenyang, China

**Keywords:** nomogram, young adults, acute ischemic stroke, unfavorable outcomes, predictive model

## Abstract

**Objective:**

This study aimed to develop and validate a nomogram for predicting the risk of 3 months adverse outcomes among young adults with acute ischemic stroke (AIS).

**Methods:**

Patients aged between 18 and 50 with acute ischemic stroke (AIS) at the Shenyang First’s People Hospital, between January 1st 2017 to May 30th 2023 were included in this retrospective study. The primary outcome was a three-month unfavorable outcome, evaluated with modified Rankin Scale (mRS > 2). Univariate logistic regression was used to select the independent factors of prognosis and multivariate logistic regression to establish a new nomogram model. We used the area under the receiver-operating characteristic curve (ROC) to evaluate the discriminative performance and used the calibration curve with Hosmer-Lemeshow goodness of fit test to assess the calibration performance of the risk prediction model. Decision curve analysis (DCA) was applied to assess the clinical utility of the nomogram.

**Results:**

A total of 1,015 patients were enrolled. Gender (male vs. female; Odds ratio[OR], 0.5562[95% Confidence Interval (CI), 0.3104–1.0478]; *p* = 0.053), family history of stroke (OR, 3.5698[95%CI 1.5632–8.0329], *p* < 0.001), prior stroke (OR, 2.1509[95%CI 1.2610–3.6577], *p* < 0.001), previous heart disease (OR, 3.4047[95%CI, 1.7838–6.6976], *p* < 0.01) toast type (cardio-embolism stroke vs. large-artery atherosclerosis (LAA), OR, 0.0847[0.0043–0.5284], *p* < 0.01), toast type (stroke of undetermined etiology vs. LAA, OR, 0.0847[0.0439–0.5284], *p* < 0.01), mRS at admission (OR, 15.2446 [9.1447–26.3156], *p* < 0.0001), adherence to medication (OR, 2.1197[95%CI, 1.1924–3.7464], *p* < 0.001), systolic blood pressure (SBP; OR, 1.0145[1.0041–1.0250], *p* < 0.001), and lactate dehydrogenase (LDH; OR, 1.0060[1.0010–1.0111], *p* < 0.01) were related to 3 months adverse outcomes among young adults with AIS. The nomogram displayed excellent calibration and discrimination. DCA confirmed the clinical applicability of the model.

**Conclusion:**

The nomogram comprised of gender, family history of stroke, prior stroke, previous heart disease, toast type, mRS score at admission, adherence to medication, SBP and LDH may predict 3 months adverse outcomes among young adults with AIS.

## Introduction

In recent years, there has been an observed increase in the occurrence of stroke among young adults aged 18 to 50 years (1, 2). Presently, it is estimated that this demographic contributes to approximately 15% of all cases of ischemic strokes (3). Despite adults having the highest survival rates, young survivors of acute ischemic stroke (AIS) may potentially experience a multitude of physical, emotional, and psychosocial challenges (4). It is crucial for patients to have a comprehensive understanding of the risk factors and causes of stroke in order to potentially mitigate future adverse outcomes, given their considerable life expectancy spanning several decades (5). By identifying potential contributors and conducting early predictions of post-stroke functional outcome, it may be possible to modify the unfavorable outcomes of strokes occurring at a young age. This approach can assist clinical physicians in adopting more beneficial therapies and enable patients to establish more reasonable expectations (6).

The nomogram is regarded as a graphical statistical tool that can generate a continuous scoring system by incorporating various variables, thereby illustrating the individual and accurate probability of risk (7, 8). The use of prognostic prediction models has been extensively researched and implemented in the assessment of various diseases, such as ischemic stroke (6).

However, a specific nomogram that accurately predicts unfavorable outcomes at 3 months following AIS in young adults has not yet been developed. Consequently, the objective of this current study is to construct a nomogram utilizing a combination of demographic factors, comorbidities, biochemical indicators, and other pertinent information to effectively forecast adverse outcomes at 3 months among young adults with AIS.

## Materials and methods

### Subjects

Data was collected retrospectively from the Shenzhou Cloud database, a stroke registry database located at Shen Yang First People’s Hospital in Shen Yang, China. This database includes consecutive patients diagnosed with AIS at a single center. The follow-up staff at the Follow-up Center of Shenyang First People’s Hospital, who have undergone professional training, conduct telephone or outpatient follow-ups with patients 3 days, 1 week, 2 weeks, 1 month, 2 months, and 3 months after discharge, recording and uploading relevant information to the database (there is no physical examination in modified Rankin Scale (mRS), so it can be assessed remotely by telephone). Patients were enrolled in the study from January 1, 2017 to May 22, 2023. The ethical review committee of Shenyang First People’s Hospital granted approval for this study [2023SYKYPZ07], all methods were performed in accordance with the relevant guidelines and regulations. All information was extracted from the database, and each AIS patient was informed about the database situation and asked for their consent (In our hospital, for clinical data, laboratory data and treatment results are used for research purposes, admitted patients are invited to give pan-informed consent and sign written informed consent). The corresponding author possesses access to all pertinent information.

The inclusion criteria encompassed the following: (1) individuals between the ages of 18 and 50, (2) diagnosed with AIS, and (3) possessing complete baseline and three-month mRS scores. The exclusion criteria were patients (1) who aged<18 or > 50 years, (2) who refused to take aspirin or statins or both after discharge, (3)who lost to be followed up in 3 months from the index date.

### Data collection

The baseline information, encompassing demographic characteristics, laboratory results, comorbidities, and other pertinent data that were all collected at the beginning of hospitalization except “adherence to medication” and “three-month outcomes,” was extracted and rectified from the database. “adherence to medication” refers to the continuous use of these two conventional medications—aspirin and statins, throughout the three-month follow-up period and the reason for using aspirin is its well-established role as a first-line antiplatelet agent in the management of AIS; the toast type was based on computer tomography (CT) imaging result or Magnetic Resonance Imaging (MRI) result, which decided and evaluated by patients’ primary care physicians before admission; “family history of stroke” refers to family members genetics related and “previous heart disease” refers to coronary heart disease, angina pectoris, myocardial infarction, heart failure, or congenital heart disease, while undiagnosed transient angina was excluded.

The Following-up Center at Shen Yang First People’s Hospital was responsible for reminding and documenting patients’ adherence to medication; the endpoint of this study was operationally defined as unfavorable outcomes, specifically with mRS scores≥3. These scores were assessed by attending physicians or following-up physicians at the three-month mark following the occurrence of the index stroke.

### Statistical analysis

The statistical tests employed for comparing continuous variables were the Student t test or the Mann–Whitney U test; the χ^2^ test or Fisher exact test was employed to compare categorical variables between two cohorts. To develop the predictive model, firstly, we divided the patients into the development and validation cohorts in a random manner, with a ratio of 3:1 (9). In addition, we employed a univariate logistic regression analyses to identify the variables selected, and those exhibiting *p* < 0.05 were subsequently integrated into a multivariate logistic regression. Furthermore, we performed a backward stepwise selection method in the development cohort to ascertain the independent prognostic factors, which subsequently led to the construction of a novel nomogram model. The discrimination was assessed by quantifying the area under the receiver operating characteristic (ROC) curve, while the calibration was evaluated through the use of a calibration curve accompanied by the Hosmer-Lemeshow goodness of fit test. Additionally, the decision curve analysis was conducted to investigate the clinical utility of the nomogram model. The analysis was conducted using the R statistical software (version 4.2.2; R Foundation for Statistical Computing, Vienna, Austria). Statistical significance was determined at a significance level of *p* < 0.05 (two-tailed).

## Results

A total of 1,015 patients, with a mean age of 44.74 ± 4.86 years and a female representation of 15%, were ultimately included in our study (Supplementary Figure S1). The occurrence of adverse outcomes over a three-month period amounted to 16%. Table 1 presented the baseline characteristics in favorable outcome cohort and adverse outcome cohort, which showed that gender was balanced even though male accounted for 85%. Supplementary Table S1 presented the baseline characteristics, which exhibited no significant differences, except for the NIHSS score upon admission and the creatine Kinase levels between the development and validation cohorts.

**Table 1:** Baseline characteristics of young adults with AIS in favorable outcome cohort and adverse outcome cohort.

	Total (*n* = 1,015)	Favorable outcome cohort (*n* = 856)	Adverse outcome cohort (*n* = 159)	*p*-value
Demographics				
Age, years	44.75 ± 4.86	44.65 ± 4.9	45.29 ± 4.62	0.116
Male, n (%)	859 (85)	730 (85)	129 (81)	0.225
Smoking, n (%)	586 (58)	503 (59)	83 (52)	0.147
Drinking, n (%)	343 (34)	295 (34)	48 (30)	0.34
DBP, mmHg	90 (80, 100)	90 (80, 100)	90 (80, 101.5)	0.232
SBP, mmHg	150 (140, 165)	150 (139, 163)	150 (140, 170)	0.059
Comorbidities				
Hypertension, n (%)	668 (66)	561 (66)	107 (67)	0.735
Heart Failure, n (%)	63 (6)	56 (7)	7 (4)	0.397
Diabetes Mellitus, n (%)	301 (30)	255 (30)	46 (29)	0.902
Previous Heart Disease, n (%)	123 (12)	84 (10)	39 (25)	<0.001
Ischemic Heart Disease, n (%)	67 (7)	41 (5)	26 (16)	<0.001
Hyperlipoidemia, n (%)	254 (25)	212 (25)	42 (26)	0.733
Family History of Stroke, n (%)	54 (5)	32 (4)	22 (14)	<0.001
Prior Stroke, n (%)	225 (22)	174 (20)	51 (32)	0.002
Atrial fibrillation, n (%)	11 (1)	8 (1)	3 (2)	0.393
Biochemical indicators				
CK, U/L	87 (61, 121.25)	87 (61.75, 121.25)	90 (56.5, 126.78)	0.785
LDH, U/L	180 (154, 199)	180 (154, 198)	180 (154.5, 203.5)	0.542
CK-MB, U/L	15.34 (12.25, 17.81)	15.3 (12.27, 17.7)	15.55 (12.18, 19.3)	0.534
Cholesterol, mmol/L	5.04 (4.3, 5.84)	5.06 (4.34, 5.85)	4.95 (4, 5.68)	0.113
HDLC, mmol/L	0.96 (0.83, 1.11)	0.96 (0.84, 1.1)	0.97 (0.81, 1.13)	0.79
LDL-C, mmol/L	3.28 (2.72, 3.83)	3.3 (2.73, 3.84)	3.22 (2.62, 3.74)	0.364
Triglyceride, mmol/L	1.77 (1.26, 2.54)	1.79 (1.26, 2.57)	1.58 (1.15, 2.29)	0.039
Total bilirubin, μmol/L	15.9 (12.2, 20.3)	15.8 (12, 20.33)	16.1 (12.45, 19.7)	0.7
Direct Bilirubin,μmol/L	2.7 (2, 3.4)	2.7 (2, 3.4)	2.86 (2, 3.7)	0.306
Total protein, g/L	69.18 (65.6, 72.8)	69.18 (65.7, 72.7)	69.18 (65, 73.35)	0.414
Albumin, g/L	43.53 (41.5, 45.9)	43.53 (41.6, 46.1)	43.3 (40.6, 45.2)	0.015
Globulin, g/L	25.6 (23, 28)	25.45 (23, 27.83)	25.63 (22.8, 28.95)	0.351
Albumin / Globulin	1.74 (1.54, 1.91)	1.75 (1.55, 1.92)	1.7 (1.48, 1.87)	0.053
ALT, U/L	23.43 (16.2, 32.94)	23.67 (16.26, 34.06)	22.97 (15.88, 29.43)	0.114
AST,U/L	20.2 (16, 24.75)	20.1 (16.2, 24.83)	20.5 (15.55, 24.15)	0.383
Creatinine, μmol/L	75 (61, 79)	74 (61, 78)	75 (58, 83.5)	0.57
BUN, mmol/L	5.27 (4.35, 5.73)	5.27 (4.36, 5.66)	5.27 (4.33, 5.97)	0.367
SUA, mmol/L	388.74 (327, 437)	388.74 (331, 437)	388.74 (318.3, 432.5)	0.369
Homocysteine, μmol/L	16.3 (11.7, 21.39)	16.3 (11.8, 21.39)	16.4 (11.35, 21.44)	0.824
Other related information				
Toast Type, n (%)				<0.001
LAA	710 (70)	576 (67)	134 (84)	
CE	17 (2)	15 (2)	2 (1)	
SAO	234 (23)	216 (25)	18 (11)	
SUE	54 (5)	49 (6)	5 (3)	
NIHSS at admission, n (%)				<0.001
<5	821 (81)	755 (88)	66 (42)	
5 ~ 15	160 (16)	88 (10)	72 (45)	
≥16	34 (3)	13 (2)	21 (13)	
MRS score at admission, n (%)				<0.001
0 ~ 2	725 (71)	688 (80)	37 (23)	
≥3	290 (29)	168 (20)	122 (77)	
ODT, hours	48 (24, 85)	48 (24, 89.25)	45 (16.5, 72)	0.107
Adherence to medication, n (%)				0.06
Yes	812 (80)	694 (81)	118 (74)	
No	203 (20)	162 (19)	41 (26)	
Treatment, n (%)				0.095
Medication	772 (76)	658 (77)	114 (72)	
Thrombolysis+ medication	36 (4)	26 (3)	10 (6)	
Endovascular+ medication	207 (20)	172 (20)	35 (22)	

CK, Creatine Kinase; LDH, lactate dehydrogenase; CK-MB, Creatine kinase isoenzymes; HDLC, high density lipid-cholesterol; LOL-C, low density lipid-cholesterol; ALT, alanine aminotransferase; AST, aspartate aminotransferase; BUN, blood urea nitrogen; SUA, Serum Uric Acid; LAA, large-artery atherosclerosis; CE: cardio-embolism; SAO: small-vessel occlusion; SOE: stroke of other determined etiology; SUE: stroke of undetermined etiology; NIHSS, National Institute of Health Stroke Scale; mRS, modified Rankin scale; ADL, activities of daily living; ODT, (stroke) onset to door time.

**Figure 1:**
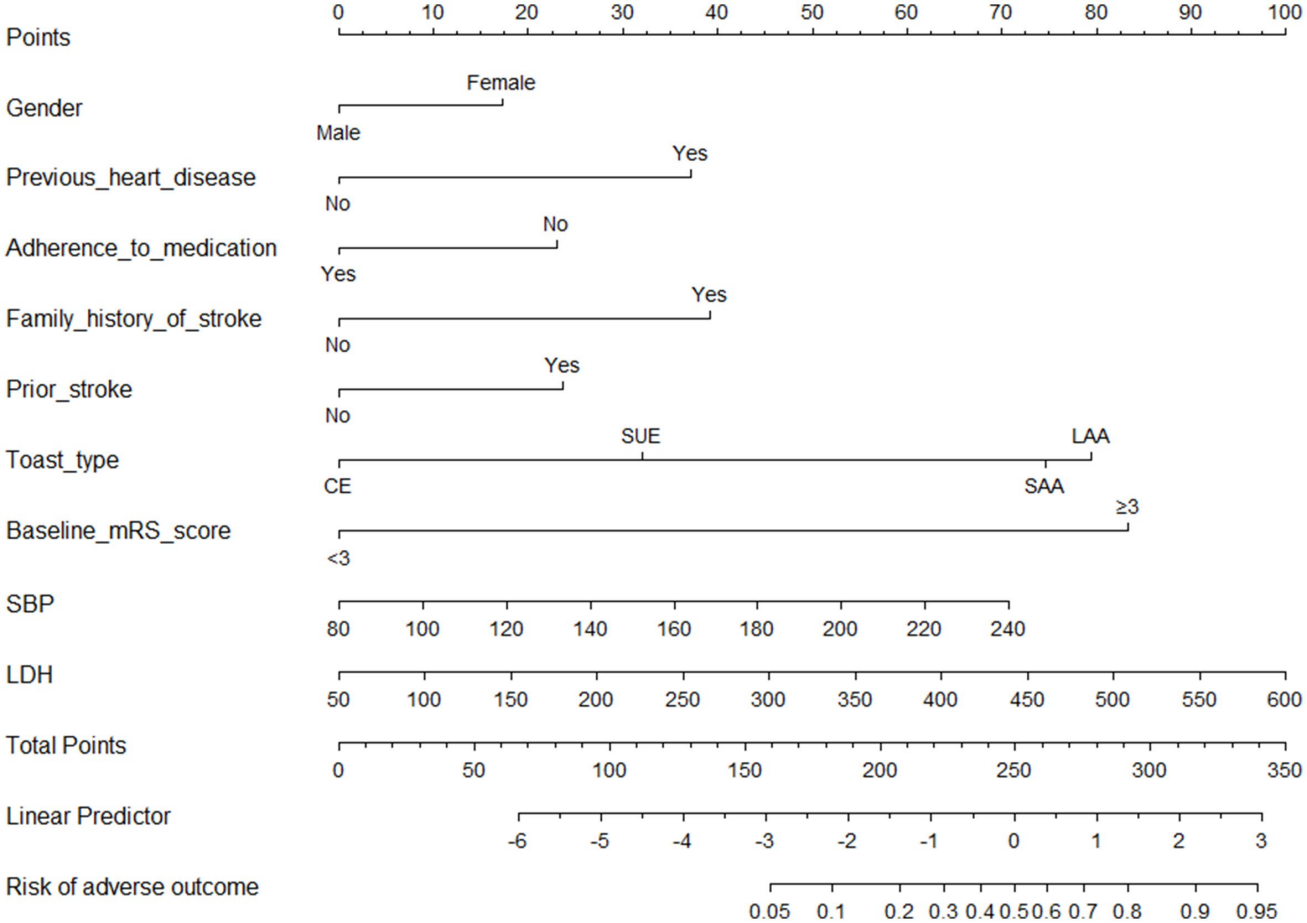
Nomogram for predicting 3 months adverse outcomes among young adults with acute ischemic stroke. LAA, large-artery atherosclerosis; CE, cardio-embolism; SAA, small-vessel occlusion; SOE, stroke of other determined etiology; SUE, stroke of undetermined etiology; MRS, modified Rankin scale; SBP indicates systolic blood pressure; LDH, lactate dehydrogenase.

The findings of the univariate logistic regression analyses were presented in Supplementary Table S2. The results presented in Table 2 indicated significant associations between various factors and the occurrence of stroke. These factors including gender (male vs. female; Odds ratio [OR], 0.5562 [95%CI, 0.3104–1.0478]; *p* = 0.053), previous heart disease (OR, 3.4047 [95%CI, 1.7838–6.6976]; *p* < 0.01), adherence to medication (OR, 2.1197 [95%CI, 1.1924–3.7464]; *p* < 0.001), family history of stroke (OR, 3.5698 [95%CI, 1.5632–8.0329]; *p* < 0.001), prior stroke (OR, 2.1509 [95%CI, 1.2610–3.6577]; *p* < 0.001), toast type (cardio-embolism stroke (CE) vs. large-artery atherosclerosis (LAA), OR, 0.0847 [0.0043–0.5284]; *p* < 0.01), and toast type (stroke of undetermined etiology (SUE) vs. LAA, OR, 0.0847 [0.0439–0.5284]; *p* < 0.01), mRS score at admission (OR, 15.2446 [9.1447–26.3156], *p* < 0.0001), systolic blood pressure (SBP; OR, 1.0145 [1.0041–1.0250], *p* < 0.001), and lactate dehydrogenase (LDH; OR, 1.0060 [1.0010–1.0111], *p* < 0.01) were determined to be significant independent risk factors for adverse outcomes at 3 months in young adults with AIS. A graphical preliminary score ranging from 0 to 100 was assigned to each of the eight predictors, and the cumulative scores were then converted into an individual probability of adverse outcomes occurring within 3 months. These probabilities were utilized to construct a novel nomogram, as depicted in Figure 1.

**Table 2:** Risk factors for 3 months adverse outcomes among young adults with AIS.

Variables	Multivariate Logistic Regression, OR (95%CI)	*p* value
Gender (male vs. female)	0.5666 (0.3106–1.0485)	*p = 0.053*
Previous Heart Disease (with vs. without)	3.3803 (1.7714–6.4491)	*p* < 0.01
Adherence to medication (yes vs. no)	2.1296 (1.1980–3.7642)	*p* < 0.001
Family history of stroke (yes vs. no)	3.6038 (1.5775–8.1106)	*p* < 0.001
Prior stroke (with vs. without)	2.1643 (1.2688–3.6806)	*p* < 0.001
Toast type		
CE vs. LAA	0.0741 (0.0038–0.4439)	*p* < 0.01
SAA vs. LAA	0.8534 (0.4065–1.7056)	*p* = 0.6627
SUE vs. LAA	0.2120 (0.0439–0.7292)	*p* < 0.01
mRS score at admission	15.2446 (9.1447–26.3156)	*p* < 0.0001
SBP	1.0145 (1.0042–1.0251)	*p* < 0.001
LDH	1.0060 (1.0010–1.0111)	*p* < 0.01

OR, odds ratio; CI, confidence interval; LAA, large-artery atherosclerosis; CE: cardio-embolism; SAA: small-vessel occlusion; SOE: stroke of other determined etiology; SUE: stroke of undetermined etiology; mRS, modified Rankin scale; SBP, systolic blood pressure; LDH, lactate dehydrogenase.

**Figure 2:**
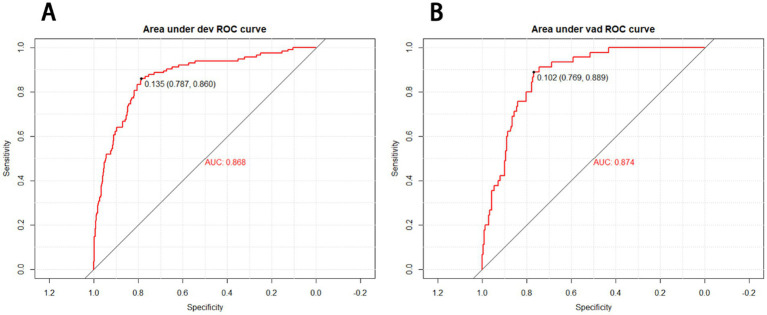
Receiver operating characteristic (ROC) curves of the nomograms in the development **(A)** and validation cohort **(B)**. The nomogram had good discriminative power with an area under the ROC curve (AUC) of 0.868 (95% CI, 0.830–0.905) and 0.874 (95% CI, 0.827–0.921) in the development and validation cohort, respectively.

**Figure 3:**
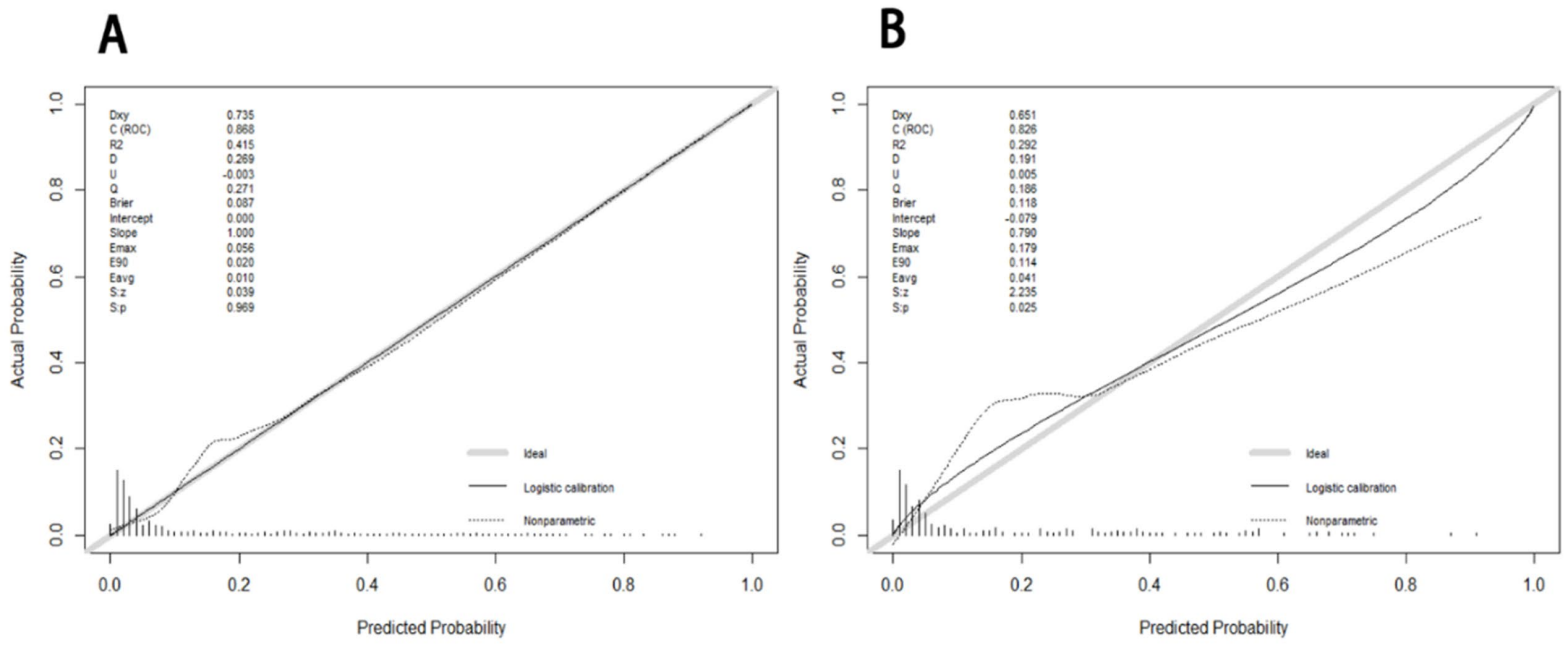
The calibration curves of nomogram for predicting 3 months adverse outcome among young adults with AIS in the development **(A)** and validation **(B)**, respectively. The calibration curves depict the calibration of the nomogram in terms of the agreement between the predicted risk of unfavorable outcomes and observed outcomes. The gray line represents a perfect prediction; the dotted lines represent the predictive performance of current nomogram and the solid line represents the predictive accuracy of the nomogram. The Hosmer-Lemeshow goodness-of-fit test is frequently used to determine whether there are any statistically significant differences between the probability of the event occurring and the actual occurrence. If *p* > 0.05, the model’s calibration was good and the calibration of this model was excellent (*p* = 0.4039).

**Figure 4:**
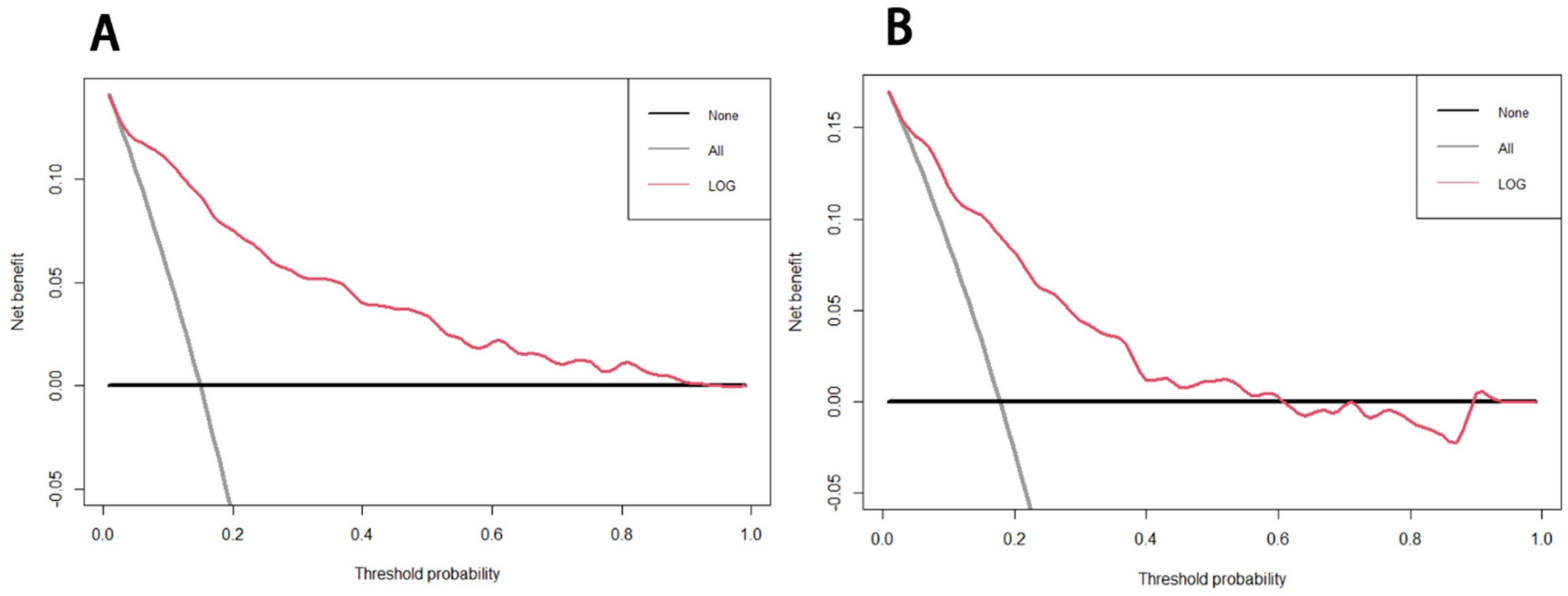
Decision curve analysis of the nomogram in the development **(A)** and validation **(B)** cohorts. The x-axis indicates the threshold probability and the y-axis represents the net benefit. The gray line displays the net benefit of the strategy of treating all patients. The black line illustrates the net benefit of the strategy of treating no patients. The red line indicates the nomogram.

The receiver operating characteristic (ROC) curves of the nomogram in the development cohort (Figure 2A) and validation cohort (Figure 2B) were presented in Figure 2, demonstrating an area under the ROC curve (AUC) of 0.868 (95% CI, 0.830–0.905) and 0.874 (95% CI, 0.827–0.921), respectively. The nomogram demonstrated high sensitivity values of 0.860 and 0.889, as well as favorable specificity values of 0.787 and 0.769, in the development and validation cohorts, respectively. Additionally, the nomogram exhibited satisfactory calibration for predicting unfavorable outcomes in young adults with AIS over a three-month period, as depicted in Figures 3A,B. Furthermore, the Hosmer-Lemeshow goodness-of-fit test yielded an excellent result (*p* = 0.4039). Decision curve analysis (DCA) was utilized to assess the clinical validity of the existing model. The inclusion of a plot (Figure 4) demonstrated a broad and pragmatic spectrum of threshold probabilities in both the development and validation cohorts, thereby indicating the model’s efficacy in forecasting adverse clinical outcomes in young patients with AIS.

## Discussion

A novel nomogram was developed incorporating gender, family history of stroke, mRS score at admission, prior stroke, toast type, previous heart disease, SBP, LDH, and adherence to medication to predict unfavorable outcomes at 3 months in young adults with AIS. The performance of this newly devised nomogram was extensively assessed and internally validated.

It has been effectively documented that factors such as female sex (10, 11), family history of stroke (genetics-related) (12, 13)and mRS score at admission≥3 (14, 15) are conventionally associated with unfavorable outcomes in patients with AIS. Our findings align with these established factors. Furthermore, we did not exclude patients with a history of prior stroke, as its recovery rates following ischemic stroke are generally benign (14). Nevertheless, our study found that it continued to be perceived as a significant predictor of unfavorable outcomes over a three-month period. Additionally, our final results also identified toast type as a conventional independent risk factor. Notably, young adults with AIS exhibited a heightened susceptibility to experiencing poor outcomes, particularly those with stroke attributed to LAA and small vessel occlusion (SAO) (16–18). According to reports, atherosclerosis serves as the underlying cause for both cardiovascular and cerebrovascular diseases, exhibiting comparable pathogenesis and etiological factors (19). Consequently, individuals with a history of heart disease may experience a heightened likelihood of unfavorable outcomes, which our findings identify as a significantly independent factor.

Three prominent predictors have captured our attention in the findings. Firstly, it has been observed that high SBP is associated with increased rates of adverse prognosis, while high blood pressure or diastolic blood pressure do not exhibit the same correlation. Furthermore, a negative correlation has been identified between the rates of unfavorable outcomes in the SBP trajectory groups and total blood pressure. In comparison to the persistently high SBP group, the groups exhibiting rapid decline, moderate-to-low, and low SBP demonstrated a reduced likelihood of unfavorable outcomes (20, 21). The present nomogram indicates that higher SB*p* values are associated with increased total points, thereby elevating the individual probability of experiencing unfavorable outcomes within a three-month period.

Additionally, the nomogram analysis revealed a significant association between elevated serum levels of LDH and unfavorable outcomes. It is important to note that LDH is primarily confined within cells unless there is local tissue injury (22). Consequently, following an acute ischemic stroke (AIS), the observed increase in serum LDH levels can be attributed to the damage or demise of brain cells. Additionally, LDH has been recognized as a noteworthy biomarker of inflammation (23), and a multitude of studies have established the pivotal role of inflammation in the pathogenesis, incidence, and prognosis of cerebral infarction (24, 25). Our findings indicate that elevated levels of LDH in serum are independently associated with an increased risk of adverse outcomes in younger stroke patients, which aligns with findings from previous scholarly articles (22, 26, 27).

Furthermore, we conducted a follow-up with young adults diagnosed with AIS to reinforce the importance of medication adherence, specifically the use of aspirin and statins. Our study revealed that approximately 80% of stroke survivors diligently adhered to the recommended low-dose aspirin regimen and cholesterol-lowering medications for a duration of 3 months. Previous research has indicated that the concurrent use of statins and aspirin is associated with reduced neurological deterioration and enhanced outcomes (28–30), making it a recommended treatment approach for patients with AIS. Our findings suggest that improved compliance with treatment regimens significantly contributes to favorable outcomes. The results of our study indicate that adherence to medication emerged as a significant independent predictor.

There is a paucity of data regarding prediction models for adverse outcomes occurring within 3 months among young adults with AIS. Previous studies that have developed scoring tools for predicting unfavorable outcomes at the three-month mark were primarily focused on the overall stroke population (6, 7, 22). Therefore, we have developed a novel nomogram that exhibits substantial discriminatory and calibration properties for the prognostication of adverse outcomes occurring within 3 months in young adults diagnosed with AIS. The occurrence of AIS with unfavorable outcomes during early adulthood has enduring consequences for individuals who find themselves at a pivotal juncture in their lives, in addition to imposing a significant socioeconomic burden. The implementation of this innovative nomogram holds the potential to facilitate the identification and monitoring of high-risk patients by healthcare professionals, enabling timely interventions that maximize patient outcomes, particularly by mitigating the likelihood of disability. Furthermore, it is imperative to implement a more assertive approach in the administration of antihypertensive therapy, anti-inflammatory medication, and neuroprotective medication, as well as conducting familial stroke screening, particularly for females. Additionally, regular cardiac examinations, strict adherence to medication, and adoption of a healthier diet should be pursued with greater vigor for this specific demographic of young adults.

Several limitations were present in this study. Firstly, it should be noted that this study was conducted retrospectively using data from a single center’s prospective database, potentially limiting its ability to accurately represent the broader population. Therefore, it is essential to conduct a multicenter validation study to verify and validate the performance of the findings. Additionally, the database does not include data on certain risk factors, such as physical inactivity, sugar blood, temperature and radiologic biomarkers like brain edema and infarct size. Moreover, the exclusion of risk factors such as diabetes mellitus, creatinine, and drinking in our study is likely to have a significant impact on the prediction model’s ability to accurately predict unfavorable outcomes. This limitation may compromise the rigor and scientific validity of the model, thereby restricting its applicability in clinical settings. Furthermore, though gender was balanced in cohorts, male was far more than female, which may not represent the overall young population fairly. However, we have conducted a supplementary sensitivity analysis regarding gender to elucidate its specific role within the model. In the construction of a new predictive model without incorporating the gender variable, utilizing the same dataset, we generated a ROC curve with an AUC value of 0.89 (95% CI, 0.8154–0.9224). Additionally, to validate the discriminatory power of this new model, we created separate ROC curves for the male and female populations, both yielding relatively high AUC values of 0.86 (95%CI, 0.8273–0.8963) and 0.9 (95% CI, 0.8438–0.9562), as shown in Supplementary Figure S2. These findings indicate that despite a significant disparity in the number of males and females, the model in this study remains robust and reliable.

Additionally, our study identified certain strengths. Notably, the absence of existing nomograms for accurately predicting adverse outcomes within a three-month timeframe rendered our findings significant. Moreover, our results exhibited a commendable level of excellence. Furthermore, it is worth mentioning that the patient screening process was conducted with utmost rigor. The primary outcome of utmost importance, specifically the three-month mRS scores of all patients, was fully obtained. Patients with incomplete mRS scores were deliberately excluded from the study, thereby ensuring the methodological rigor and scientific validity of the predictive model, and facilitating its broad applicability in clinical settings. Patients who lost to be followed up were excluded, which minimized the follow-up bias.

## Conclusion

In conclusion, a novel nomogram was developed that incorporates various factors such as gender, family history of stroke, mRS score at admission, prior stroke, toast type, SBP, previous heart disease, LDH, and adherence to medication. This nomogram effectively predicts three-month unfavorable outcomes for young adults with AIS. However, additional external validations are necessary to assess the applicability and reliability of this model in broader contexts.

## Data Availability

The raw data supporting the conclusions of this article will be made available by the authors, without undue reservation.
