# Supplementary material for: Nomogram to predict 3 month prognosis of acute ischemic stroke among young adults
Source: Front Neurol. 2025 Jan 30;15:1487248. doi: 10.3389/fneur.2024.1487248 (PMC11822686; doi:10.3389/fneur.2024.1487248)
Supplement: Supplementary file 1 [file Data_Sheet_1.PDF]

Supplemental data:

Supplemental Table 1

Baseline characteristics of young adults with AIS in development cohort and validation cohort.

	Total (n=1015)	development cohort (n=762)	validation cohort (n=253)	*P*-value
**Demographics**				
Age, years	44.75±4.86	44.76±4.87	44.72±4.84	0.909
Gender, n(%)				0.137
Male	859 (85)	637 (84)	222 (88)	
Female	156 (15)	125 (16)	31 (12)	
Smoking, n(%)				0.093
Yes	586 (58)	428 (56)	158 (62)	
No	429 (42)	334 (44)	95 (38)	
Drinking, n(%)				0.22
Yes	343 (34)	249 (33)	94 (37)	
No	672 (66)	513 (67)	159 (63)	
DBP, mmHg	90 (80, 100)	90 (80, 100)	90 (80, 101)	0.371
SBP, mmHg	150 (140, 165)	150 (138, 165)	150 (140, 170)	0.298
**Comorbidities**				
Hypertension, n(%)				0.879
Yes	668 (66)	500 (66)	168 (66)	
No	347 (34)	262 (34)	85 (34)	
Heart Failure, n(%)				0.811
Yes	63 (6)	46 (6)	17 (7)	
No	952 (94)	716 (94)	236 (93)	
Diabetes Mellitus, n(%)				0.808
Yes	301 (30)	228 (30)	73 (29)	
No	714 (70)	534 (70)	180 (71)	
Previous Heart Disease, n(%)				0.797
Yes	123 (12)	94 (12)	29 (11)	
No	892 (88)	668 (88)	224 (89)	
Ischemic Heart Disease, n(%)				0.953
Yes	67 (7)	51 (7)	16 (6)	
No	948 (93)	711 (93)	237 (94)	
Hyperlipoidemia, n(%)				0.638
Yes	254 (25)	194 (25)	60 (24)	
No	761 (75)	568 (75)	193 (76)	
Family History of Stroke, n(%)				0.526
Yes	54 (5)	43 (6)	11 (4)	
No	961 (95)	719 (94)	242 (96)	
Prior Stroke, n(%)				0.919
Yes	225 (22)	170 (22)	55 (22)	
No	790 (78)	592 (78)	198 (78)	
Atrial fibrillation, n (%)				0.309
Yes	11 (1)	10 (1)	1 (0)	
No	1004 (99)	752 (99)	252 (100)	
**Biochemical indicators**				
CK, U/L	87 (61, 121.25)	85.1 (60, 121.25)	96 (64, 132)	0.043
LDH, U/L	180 (154, 199)	180.1 (155, 199.07)	178 (152.1, 199)	0.685
CK-MB, U/L	15.34 (12.25, 17.81)	15.27 (12.06, 17.75)	15.57 (12.57, 18)	0.437

Cholesterol, mmol/L	5.04 (4.3, 5.84)	5.04 (4.3, 5.84)	5.05 (4.35, 5.83)	0.669
HDLC, mmol/L	0.96 (0.83, 1.11)	0.96 (0.84, 1.11)	0.96 (0.81, 1.09)	0.352
LDL-C, mmol/L	3.28 (2.72, 3.83)	3.28 (2.7, 3.84)	3.27 (2.78, 3.81)	0.552
Triglyceride, mmol/L	1.77 (1.26, 2.54)	1.78 (1.24, 2.54)	1.73 (1.3, 2.51)	0.872
Total bilirubin, μmol/L	15.9 (12.2, 20.3)	15.9 (12, 20.08)	16 (12.4, 20.9)	0.501
Direct Bilirubin ,μmol/L	2.7 (2, 3.4)	2.7 (2, 3.4)	2.7 (2, 3.4)	0.99
Total protein, g/L	69.18 (65.6, 72.8)	69.18 (65.5, 73)	69.18 (65.9, 72.5)	0.718
Albumin , g/L	43.53 (41.5, 45.9)	43.53 (41.4, 45.9)	43.53 (41.6, 45.7)	0.459
Globulin, g/L	25.6 (23, 28)	25.5 (23, 28.1)	25.63 (23, 27.7)	0.74
Albumin / Globulin	1.74 (1.54, 1.91)	1.74 (1.52, 1.91)	1.75 (1.57, 1.95)	0.314
ALT, U/L	23.43 (16.19, 32.94)	23.22 (15.74, 33.46)	23.9 (16.77, 31.38)	0.581
AST,U/L	20.2 (16, 24.75)	20.05 (16, 24.5)	20.8 (16.1, 25.2)	0.454
Creatinine, μmol/L	75 (61, 79)	74 (60, 79)	75.54 (63, 78)	0.52
BUN, mmol/L	5.27 (4.35, 5.73)	5.27 (4.39, 5.74)	5.24 (4.24, 5.66)	0.219
SUA, mmol/L	388.74 (327, 437)	388.74 (327, 440.75)	388.74 (329, 429)	0.889
Homocysteine, μmol/L	16.3 (11.7, 21.39)	16.55 (11.7, 21.39)	16 (11.8, 21.5)	0.902
**Other related information**				
Toast Type, n(%)				0.996
LAA	710 (70)	534 (70)	176 (70)	
CE	17 (2)	13 (2)	4 (2)	
SAA	234 (23)	174 (23)	60 (23)	
SUE	54 (5)	41 (5)	13 (5)	
NIHSS at admission, n (%)				0.033
<5	821 (81)	629 (83)	192 (76)	
5~15	160 (16)	107 (14)	53 (21)	
⩾16	34 (3)	26 (3)	8 (3)	
MRS score at admission, n (%)				0.187
0~2	725 (71)	553 (73)	172 (68)	
⩾3	290 (29)	209 (27)	81 (32)	
ODT, hours	48 (24, 85)	48 (24, 90)	48 (24, 72)	0.584
Adherence to medication, n (%)				0.87
Yes	812 (80)	611 (80)	201 (79)	
No	203 (20)	151 (20)	52 (21)	
Treatment, n(%)				0.82
Medication	772(76)	583(77)	189(75)	
Thrombolysis+ medication	36(4)	27(4)	9(4)	
Endovascular+ medication	207(20)	152(20)	55(22)	
Three-month outcomes, n (%)				0.331
mRS<3	856 (84)	648 (85)	208 (82)	
mRS⩾3	159 (16)	114 (15)	45 (18)	

Abbreviations: CK, Creatine Kinase; LDH, lactate dehydrogenase; CK-MB, Creatine kinase isoenzymes; HDLC, high density lipid-cholesterol; LOL-C, low density lipid-cholesterol; ALT, alanine aminotransferase; AST, aspartate aminotransferase; BUN, blood urea nitrogen; SUA, Serum Uric Acid; LAA, large-artery atherosclerosis; CE: cardio-embolism; SAA: small-vessel occlusion; SOE: stroke of other determined etiology; SUE: stroke of undetermined etiology; NIHSS, National Institute of Health Stroke Scale; mRS, modified Rankin scale; ADL, activities of daily living; ODT, (stroke) onset to door time.

Supplemental Table 2

Univariate Analysis of the Risk Factors for three months Adverse Outcomes among Young Adults with AIS.

Characteristics	OR	CI	P
Gender(male vs female)	0.61	0.38-0.99	0.05
Smoking(yes vs no)	0.72	0.48-1.07	0.1
Heart disease(yes vs no)	0.84	0.35-2.04	0.71
Drinking(yes vs no)	0.66	0.42-1.04	0.08
Previous heart disease(yes vs no)	3.06	1.87-5.01	P<0.01
Diabetes mellitus(yes vs no)	0.81	0.52-1.27	0.36
Hypertension(yes vs no)	1.11	0.72-1.69	0.64
Atrial fibrillation(yes vs no)	1.43	0.3-6.82	0.65
Adherence to medication(yes vs no)	1.83	1.17-2.87	0.01
ischemic heart disease(yes vs no)	4.23	2.32-7.74	P<0.01
Hyperlipoidemia(yes vs no)	1.05	0.67-1.66	0.82
Family history of stroke(yes vs no)	3.35	1.73-6.5	P<0.01
Prior stroke(yes vs no)	2.26	1.47-3.47	P<0.01
Toast types			
CA vs LAA	0.46	0.06-3.6	0.46
SAO vs LAA	0.4	0.22-0.72	P<0.01
SUE vs LAA	0.37	0.11-1.22	0.1
SOE vs LAA	0	0-Inf	0.98
MRS score at admission(≥3 vs <3 scores）	13.11	8.2-20.96	P<0.01
NIHSS at admission,(scores)			
5~15 vs <5	8.51	5.3-13.68	P<0.01
≥16 vs <5	12.68	5.58-28.81	P<0.01
Treatment			
Thrombolysis +medication VS medication	1.59	0.62-4.04	0.33
Endovascular +medication VS medication	0.75	0.43-1.28	0.29
Age	1.03	0.98-1.07	0.2
ODT, (hours)	1.0	1.0-1.0	0.77
DBP, (mmHg)	1.01	0.99-1.02	0.33
SBP, (mmHg)	1.01	1-1.02	0.02
CK, (U/L)	1.0	1.0-1.0	0.95
LDH, (U/L)	1.01	1-1.01	P<0.01
CK-MB, (U/L)	1.02	0.99-1.04	0.22
CHOL,(mmol/L)	0.89	0.76-1.04	0.13
HDLC, (mmol/L)	1.1	0.57-2.11	0.78
LDL-C,(mmol/L)	0.97	0.76-1.23	0.8
TG, (mmol/L)	0.92	0.77-1.1	0.35
TBIL,(μmol/L)	1.01	0.98-1.03	0.49
DBIL,(μmol/L)	1.1	0.96-1.26	0.16
TP,(g/L)	0.99	0.95-1.02	0.38
ALB,(g/L)	0.95	0.9-0.99	0.02
GLOB, (g/L)	1.02	0.98-1.07	0.35
ALB/GLOB	0.78	0.43-1.42	0.42
ALT, (U/L)	0.99	0.98-1.01	0.32
AST,(U/L)	1.0	0.98-1.01	0.91
Creatinine,(μmol/L)	1.0	1-1.01	0.06
BUN, (mmol/L)	0.98	0.87-1.1	0.69
SUA, (mmol/L)	1.0	1.0-1.0	0.20

Homocysteine,(μmol/L)	1.0	0.99-1.01	0.68

Abbreviations: OR, odds ratio; CI, confidence interval ;CK, Creatine Kinase; LDH, lactate dehydrogenase; CK-MB, Creatine kinase isoenzymes; HDLC, high density lipid-cholesterol; LOL-C, low density lipid-cholesterol; ALT, alanine aminotransferase; AST, aspartate aminotransferase; BUN, blood urea nitrogen; SUA, Serum Uric Acid; LAA, large-artery atherosclerosis; CE: cardio-embolism; SAO: small-vessel occlusion; SOE: stroke of other determined etiology; SUE: stroke of undetermined etiology; NIHSS, National Institute of Health Stroke Scale; mRS, modified Rankin scale; ADL, activities of daily living; ODT, (stroke) onset to door time.

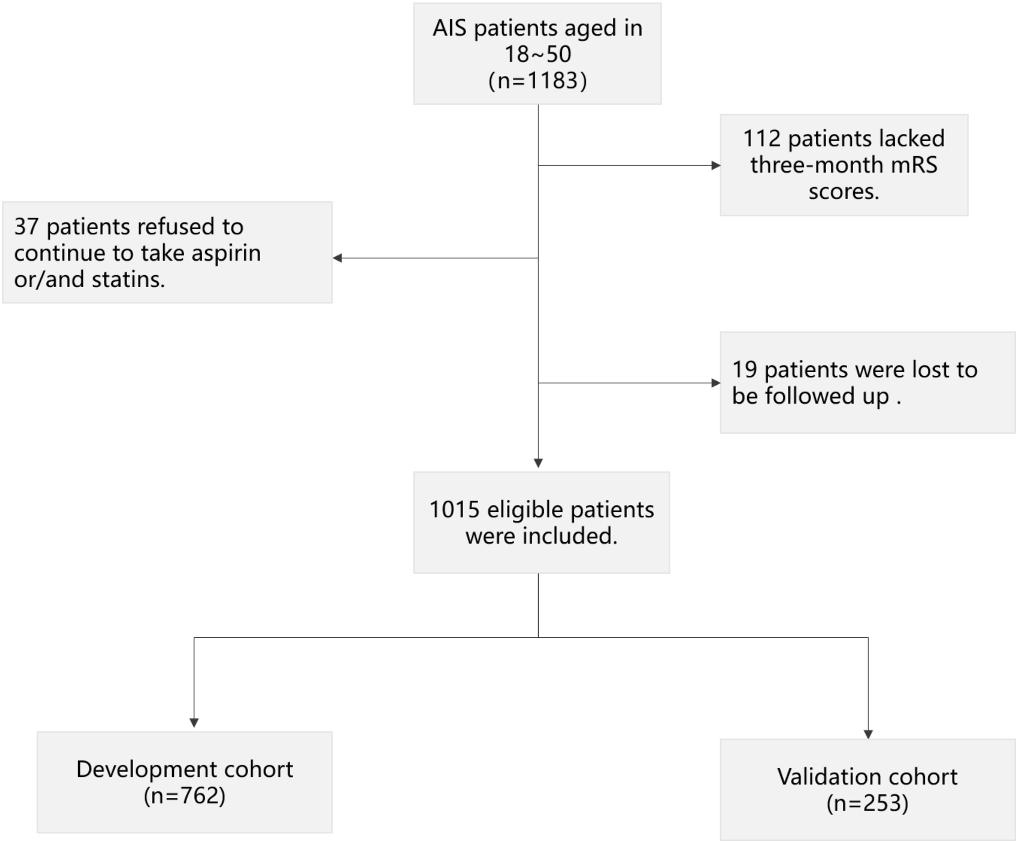

Fig S1: Flowchart for screening participants.

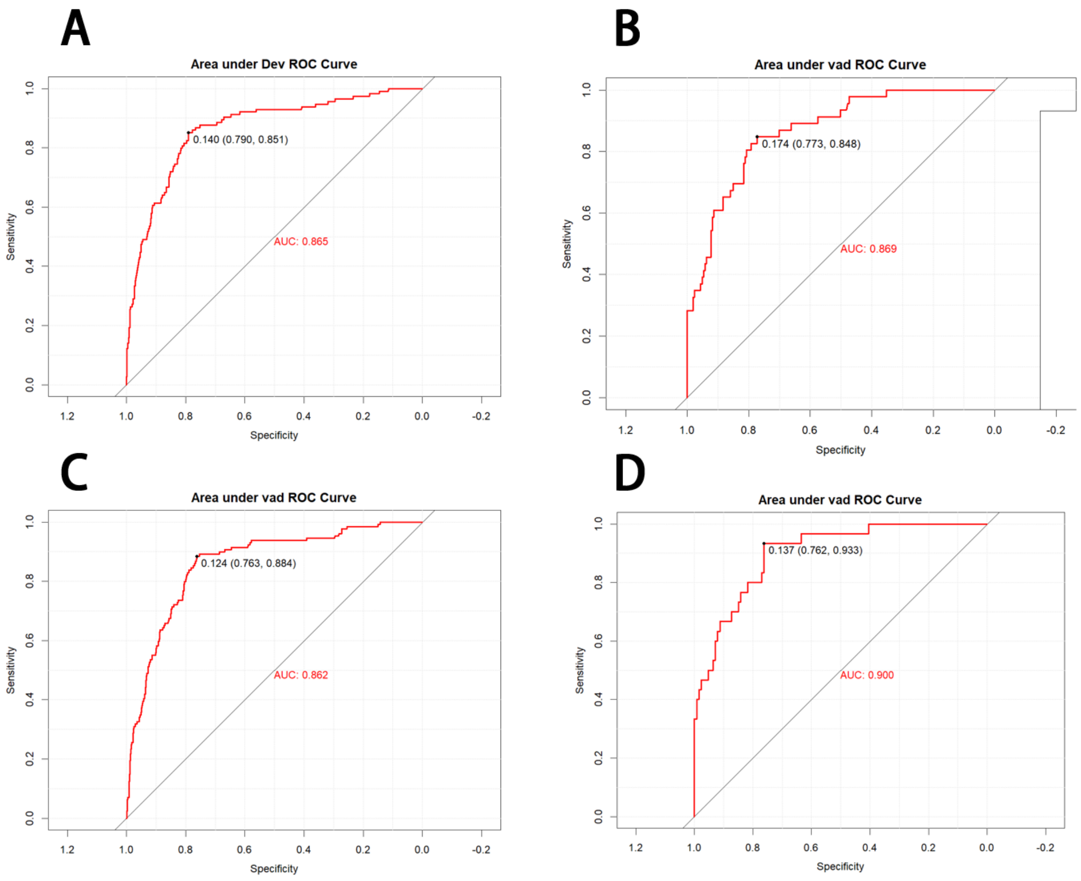

Fig S2: Receiver operating characteristic (ROC) curves of the new prediction model using the same population without gender variable in the development(A) and validation cohort(B); using the male population(C) and female population (D) to validate the discriminatory power of the new model.
